# Antitumor effect of 4MU on glioblastoma cells is mediated by senescence induction and CD44, RHAMM and p-ERK modulation

**DOI:** 10.1038/s41420-021-00672-0

**Published:** 2021-10-09

**Authors:** Matías Arturo Pibuel, Daniela Poodts, Mariángeles Díaz, Yamila Azul Molinari, Paula Gabriela Franco, Silvia Elvira Hajos, Silvina Laura Lompardía

**Affiliations:** 1 Instituto de Estudios de la Inmunidad Humoral (IDEHU)- CONICET; Departamento de Microbiología, Inmunología y Biotecnología, Facultad de Farmacia y Bioquímica, Universidad de Buenos Aires, Capital Federal, Argentina; 2 Instituto de Estudios de la Inmunidad Humoral (IDEHU)- CONICET, Universidad de Buenos Aires, Capital Federal, Argentina; 3 Instituto de Química y Fisicoquímica Biológicas (IQUIFIB)-CONICET; Departamento de Química Biológica, Cátedra de Química Biológica Patológica, Facultad de Farmacia y Bioquímica, Universidad de Buenos Aires, Capital Federal, Argentina

**Keywords:** Chemotherapy, CNS cancer, Senescence

## Abstract

The extracellular matrix plays a key role in cancer progression. Hyaluronan, the main glycosaminoglycan of the extracellular matrix, has been related to several tumor processes. Hyaluronan acts through the interaction with cell membrane receptors as CD44 and RHAMM and triggers signaling pathways as MEK/ERK. 4-methylumbelliferone (4MU), a well-known hyaluronan synthesis inhibitor, is a promising alternative for cancer therapy. 4MU is a coumarin derivative without adverse effects that has been studied in several tumors. However, little is known about its use in glioblastoma (GBM), the most malignant primary brain tumor in adults. Glioblastoma is characterized by fast growth, migration and tissue invasiveness, and a poor median survival of the patients after treatment. Several reports linked glioblastoma progression with HA levels and even with CD44 and RHAMM expression, as well as MEK/ERK activation. Previously, we showed on a murine GBM cell line that HA enhances GBM migration, while 4MU markedly inhibits it. In this work we showed for the first time, that 4MU decreases cell migration and induces senescence in U251 and LN229 human GBM cell lines. Furthermore, we observed that HA promotes GBM cell migration on both cell lines and that such effects depend on CD44 and RHAMM, as well as MEK/ERK signaling pathway. Interestingly, we observed that the exogenous HA failed to counteract the effects of 4MU, indicating that 4MU effects are independent of HA synthesis inhibition. We found that 4MU decreases total CD44 and RHAMM membrane expression, which could explain the effect of 4MU on cell migration. Furthermore, we observed that 4MU increases the levels of RHAMM inside the cell while decreases the nucleus/cytoplasm relation of p-ERK, associated with 4MU effects on cell proliferation and senescence induction. Overall, 4MU should be considered as a promising therapeutic alternative to improve the outcome of patients with GBM.

## Introduction

The impact of tumor microenvironment on cancer progression has become of great interest. The extracellular matrix (ECM) plays an active role in cell proliferation, death evasion, angiogenesis, migration, invasion, immune suppression, and multidrug resistance in the tumor context [1, 2].

Hyaluronan (HA) is the main glycosaminoglycan (GAG) of the ECM and the key component of brain parenchymal ECM. A precise balance between HA synthesis, degradation, and internalization determines HA levels [3–7]. In a cancer context, when the quantity of HA exceeds the physiological levels and/or when its quality is altered, tumor progression-related processes are enhanced [4, 8–11]. These effects are exerted through HA binding to its receptors, such as CD44 and RHAMM, and the triggering of several signaling pathways involved in tumor progression [12]. In this way, HA-CD44 as well as HA-RHAMM interactions, can activate MEK which mediates ERK phosphorylation, promoting cell migration and survival in different malignancy models [12–15].

Considering the impact of HA in cancer progression, many strategies have been developed to mitigate its effects [9]. In this way, 4-methylumbelliferone (4MU), a coumarin derivative without toxic effects, approved to be used as an antispasmodic and choleretic agent in Europe and Asia [16, 17] has been used as HA-synthesis inhibitor [17, 18]. Furthermore, 4MU abrogates the proliferation and migration of several tumor cells [13, 16, 17, 19–24] and some effects of 4MU would be independent of HA synthesis inhibition, such as modulation of metalloproteinases (MMPs) activity and CD44 expression [24, 25]. Although 4MU has been studied on several malignancies, little is known about its effects on glioblastoma (GBM) [26, 27].

GBM is the most malignant primary brain tumor in adults. Also known as IV grade glioma, it is characterized by fast growth, migration, and tissue invasiveness [28]. Although new data obtained of phase III clinical trials indicate some improvement in the overall survival time [29], most of the data available showed that under the current therapy that includes surgical resection, radiotherapy, and treatment with temozolomide (TMZ), the median survival of the patients is only 14.6 months [30–32].

Therefore, the study and development of new therapeutic alternatives are necessary. Several reports linked glioblastoma progression with HA levels and, as described in our recent review, hyaluronan enhances proliferation and migration of GBM cells [33–35]. Moreover, it was demonstrated that RHAMM enhances the invasiveness of GBM cells, while the expression of CD44 has been correlated with decreased survival in patients as well as the increase of cell proliferation, invasion, and chemoresistance in GBM cells [36–39]. Finally, MMPs have also been associated with poor prognosis in GBM [40, 41].

Taking into account this background, we hypothesized that HA promotes GBM progression and, therefore, 4MU would be a potential new drug for GBM therapy, not only due to its known effect on HA synthesis but also through independent mechanisms.

Our results showed for the first time that 4MU markedly inhibits cell migration and induces senescence in human GBM cell lines. We also found that 4MU acts by modulating the expression and the distribution of CD44, RHAMM, MMP-2, and p-ERK on human GBM cell lines. These findings highlight the potential use of 4MU for GBM treatment.

## Results

### LMW-HA and HMW-HA enhance cell migration in both cell lines

Considering that fast growth and migration are involved in GBM progression, we evaluated if HA modulates such biological processes. U251 and LN229 cells were treated with HMW-HA or LMW-HA and XTT, BrdU incorporation, wound-healing as well as zymography assays were performed.

**Fig. 1:**
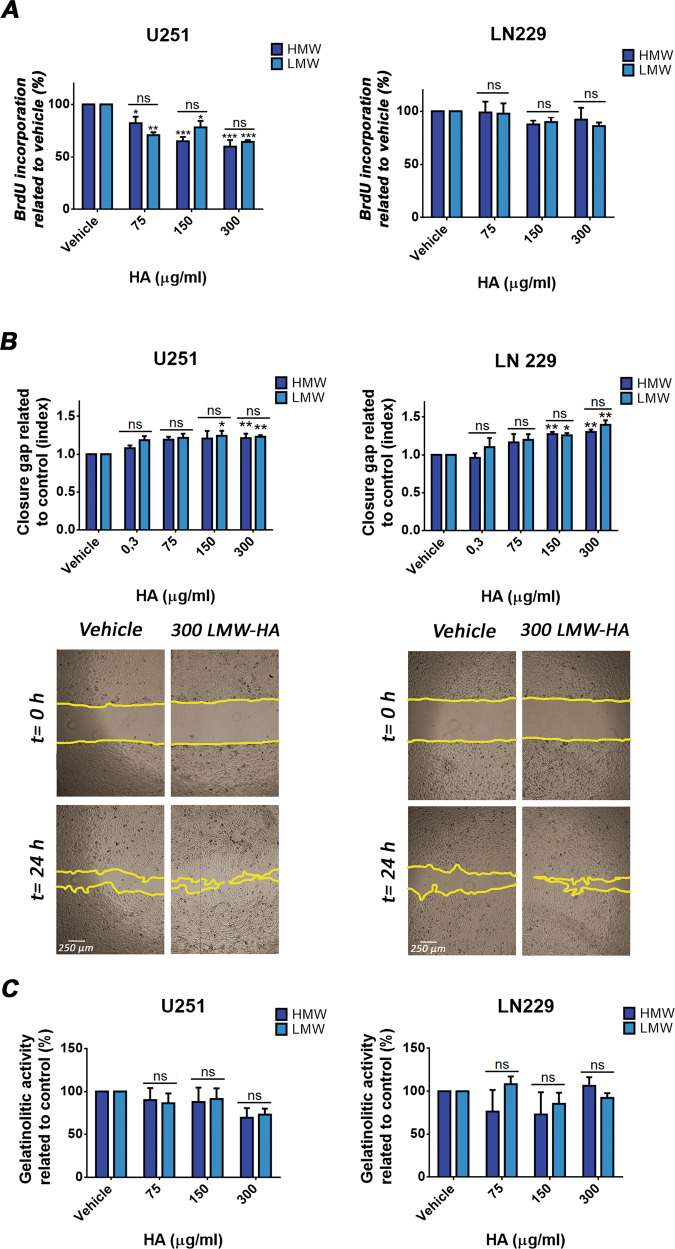
Effect of HA on cell proliferation and cell migration. **A** Cell proliferation was determined by BrdU incorporation, ELISA-like and immunofluorescence assays after 48 h of treatment with HMW-HA and LMW-HA. Results are expressed as the percentage of cell proliferation (*n* = 3) in relation to vehicle control cells as described in the Materials and Methods section. **B** U251 and LN229 cell lines were treated with either HMW-HA or LMW-HA and cell migration was determined by the wound healing assay after 24 h of treatment. The same wound area was photographed at 0 and 24 h. Results are expressed as closure gap index (*n* = 3) calculated as described in Material and Methods. Representative images of independent assays are shown under each bar graph (magnification: ×40). **C** MMPs activity of U251 and LN229 cells after 24 h of treatment determined by zymography. The gelatinolytic activity was calculated as the percentage of densitometry values (*n* = 4) of bands in relation to vehicle control cells. In all graphs, bars represent means ± SEM of at least three independent experiments. Asterisks over bars indicate differences between treated and cells treated with vehicle, * = *p* < 0.05 and ** = *p* < 0.01, ns= non-significant (*p* > 0.05). Symbols over lines indicate differences between the indicated treatments. Asterisks represent statistical significance in relation to cells treated with vehicle.

Unexpectedly, we found that both HMW-HA and LMW-HA inhibited cell proliferation on U251 cells and did not modify this process on LN229 cell line after 48 h of treatment (Fig. 1A). Similar results were obtained by XTT assay on LN229 cells while 300 µg/ml HA augmented the metabolic activity on U251 cell line (Fig. Sup. 1).

Interestingly, both HMW-HA and LMW-HA enhanced wound closure in LN229 and U251 cells (Fig. 1B), without modifying MMP-2 activity (Fig. 1C).

Overall, HA enhances GBM cell migration without affecting MMP-2 activity. Furthermore, HA increased metabolic activity but reduced cell proliferation on U251 cells while it did not modify such processes on LN229 cells.

### HA-induced migration is mediated by CD44, RHAMM and MEK

We next explored if CD44, RHAMM, and MEK/ERK were involved in HA-enhanced migration in U251 and LN229 cells. As shown in Fig. 1A the 300 μg/ml HA dose stimulated the migration most strongly, and HMW-HA and LMW-HA similarly stimulated cell migration on these cells. Considering that together with previous works that reported that LMW-HA was more relevant than HMW-HA in such process [42, 43], we decided to use 300 μg/ml LMW-HA to perform the assays. For a correct interpretation of the results, we select a dose of antibodies and U0126 inhibitor that did not modify the migration per se at the times of these assays.

**Fig. 2:**
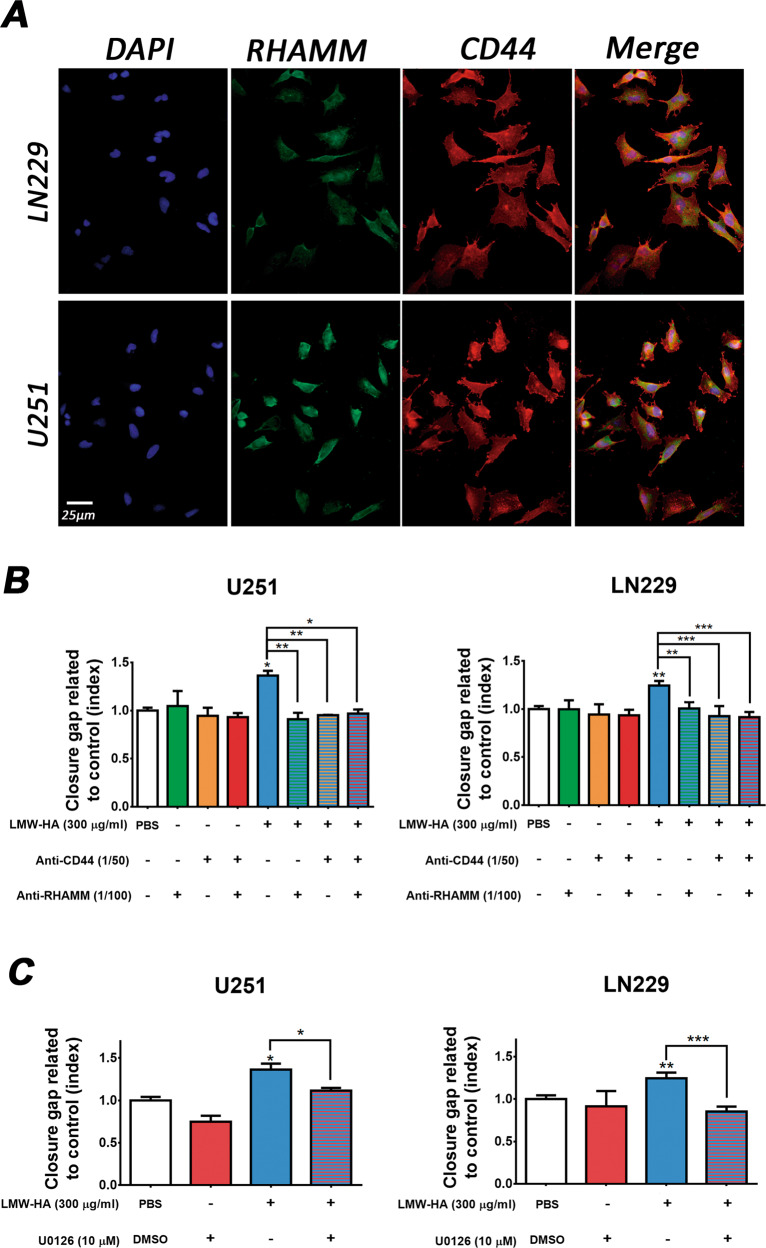
Implication of RHAMM, CD44 and MEK in HA-enhanced migration. **A** Expression of RHAMM and CD44 on both LN229 (top) and U251 (bottom) was evaluated by IF assay (magnification: ×200). U251 (left) and LN229 (right) cells were treated with anti-RHAMM or/and anti-CD44 antibodies (**B**) or with U0126 inhibitor (**C**) for 24 h and wound healing assay was performed. The same wound area was photographed at 0 and 24 h. Results are expressed as closure gap index calculated as described in the Material and Methods section. In all graphs, bars represent means ± SEM of three independent experiments. Asterisks represent statistical significance in relation to cells treated with vehicle: * = *p* < 0.05 and ** = *p* < 0.01, *** = *p* < 0.001. Symbols over lines indicate differences between the indicated treatments.

First, the expression of CD44 and RHAMM was determined on both studied cells. As shown in Fig. 2A, U251 and LN229 cells expressed both receptors.

Figure 2B shows that the effect of HA on wound closure in both U251 and LN229 cell lines is counteracted by the addition of blocking anti-CD44, as well as anti-RHAMM antibodies. Similarly, the inhibition of MEK with U0126 abrogated the effect of HA in both cell lines (Fig. 2C).

These results indicated that RHAMM and CD44, as well as MEK activity are involved in HA-induced migration in both GBM cell lines.

### 4MU decreases HA synthesis and markedly inhibits cell proliferation but induces low levels of cell death on GBM cells

As 4MU is a well-known HA-synthesis inhibitor and considering that HA enhances GBM malignant features, we decided to investigate the effect of 4MU on HA synthesis by ELISA-like assay, cell growth by BrdU incorporation, and cell death by FC staining with PI.

**Fig. 3:**
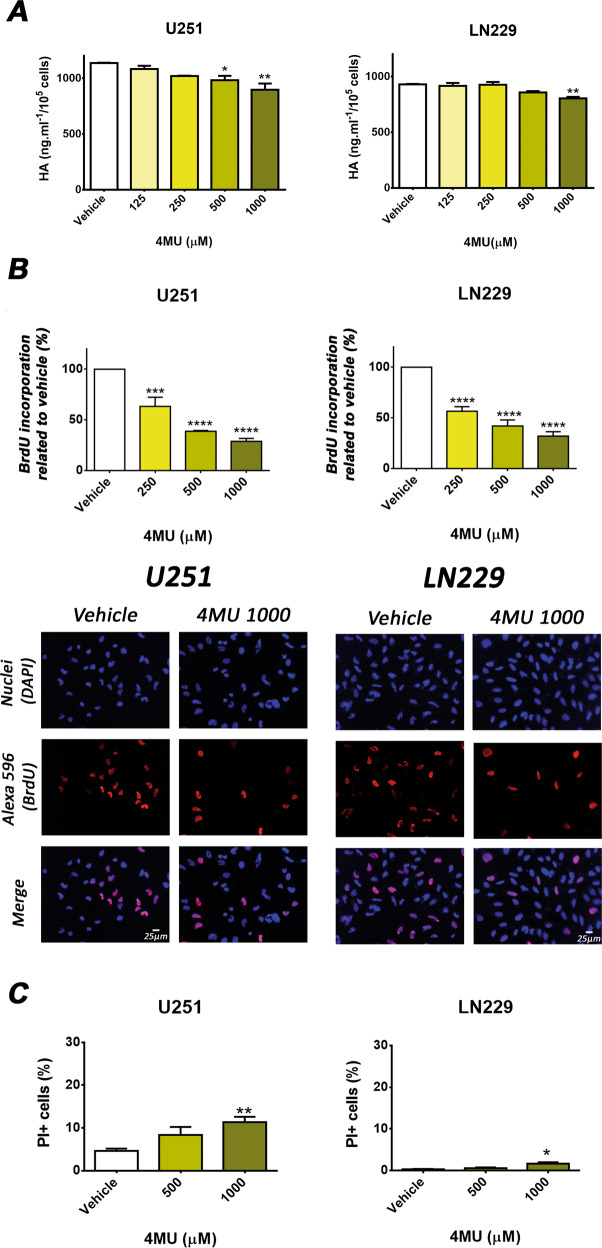
Effect of 4MU on hyaluronan synthesis, cell proliferation and cell death. **A** Levels of HA in U251 and LN229 culture supernatants were evaluated by ELISA after 24 h of treatment with 4MU. Values are expressed as HA concentration (ng/ml/10^5^cells) (*n* = 3). **B** Cell proliferation was determined by BrdU incorporation and ELISA-like as well as immunofluorescence assays after 48 h of treatment. Results are expressed as the percentage (*n* = 4) of cell proliferation in relation to vehicle control cells as described in the Material and Methods section. Representative IF staining is shown under each bar graph (magnification: ×200). **C** Cell death was evaluated by FC using FDA/PI stain after treatment with 4MU for 72 h. The FDA stain was used as viability control. Bars represent the percentage (*n* = 4) of PI-positive cells. Data are expressed as the mean ± SEM of at least three independent experiments. Asterisks represent statistical significance with respect to cells treated with vehicle: * = *p* < 0.05, ** = *p* < 0.01, *** = *p* < 0.001 and **** = *p* < 0.0001.

Figure 3A shows that both U251 and LN229 cells synthesized HA (1137 ± 4.61 ng/ml and 929.2 ± 5.62 ng/ml, respectively), while 4MU reduced HA synthesis at the higher dose assessed. Similar results were obtained for 1000 µM 4MU by Yan et al. [27].

Moreover, cell proliferation was reduced in a dose-dependent manner in both U251 and LN229 cells after 4MU treatment. It is noteworthy that at the highest dose assessed, cell proliferation was inhibited about 70% in both cell lines (Fig. 3B). Furthermore, this drug increased the number of PI-positive cells. However, at the highest 4MU dose of 1000 µM the percentages reached were only about 15% and 5% in the U251 and LN229 cell lines, respectively (Fig. 3C). It is worth noting that we have previously demonstrated the inhibition of metabolic activity by 4MU in a dose-dependent manner in both cell lines [26].

These results suggest that, although 4MU markedly reduced cell proliferation, it failed to induce high percentages of cell death on GBM cells.

### 4MU induces senescence in both cell lines

Given that 4MU markedly inhibited cell proliferation but induced low levels of cell death, we evaluated the effect of 4MU on senescence induction, another tumor-suppressive mechanism. For this purpose, the activity of senescence-associated β-galactosidase (SA-β-gal) and the presence of cytoplasmic chromatin fragments (CCF) were determined.

**Fig. 4:**
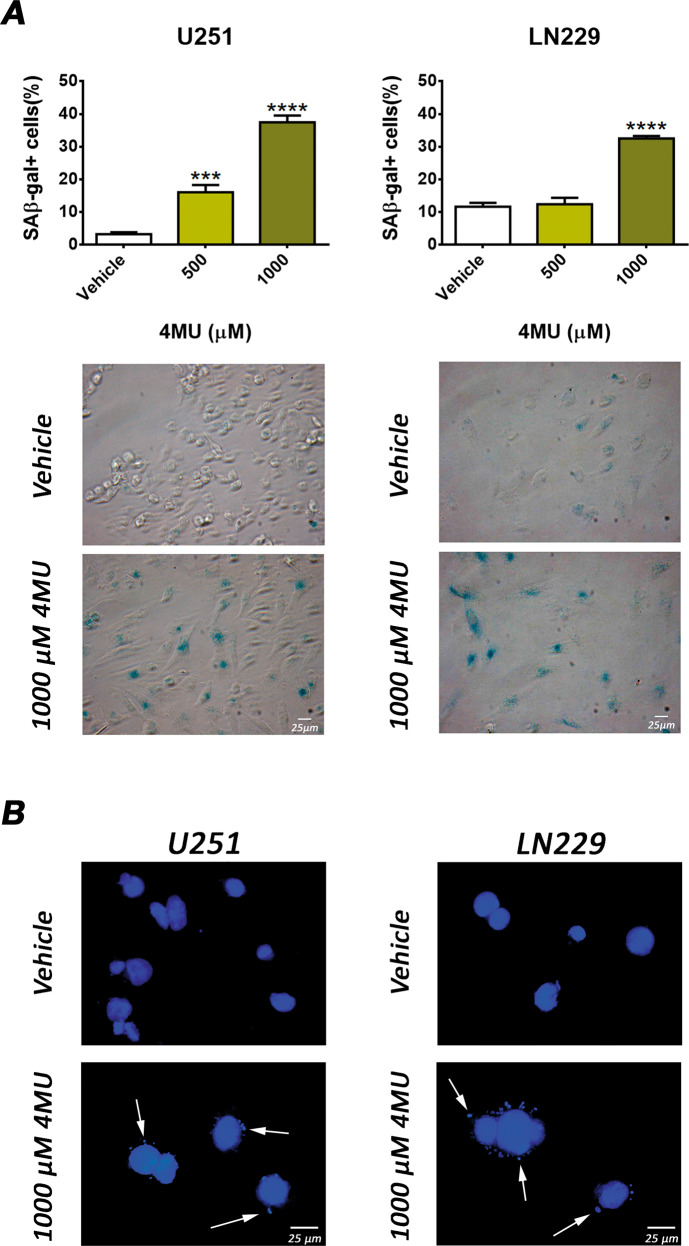
Effect of 4MU on senescence induction. **A** U251 and LN229 cell lines were treated with 4MU for 48 h and senescence induction was evaluated by SA-β-gal activity assay. Results are expressed as the percentage (*n* = 3) of SA-β-gal positive cells in relation to vehicle control as described in the Material and Methods section. Representative images of independent assays are shown under each bar graph (magnification: ×200). **B** The presence of CCFs was determined by DAPI staining after 48 of treatment with 4MU in U251 (left) and LN229 (right). Representative pictures of independent assays (*n* = 3) are shown under each bar graph (maginification: ×400). Arrows show CCF in treated cells. Data are expressed as the mean ± SEM of three independent experiments. Asterisks represent statistical significance with respect to cells treated with vehicle: *** = *p* < 0.001 and **** = *p* < 0.0001.

As shown in Fig. 4A, 4MU increased the percentage of SA-β-gal positive cells in both LN229 and U251 cell lines. Likewise, CCF were observed after treatment of both cell lines with 4MU (Fig. 4B).

These results indicate that 4MU enhanced the induction of senescence in both cell lines.

### 4MU decreases cell migration and MMP-2 activity in LN229 and U251 cells

As cell migration and MMP activity are widely involved in GBM progression, it was relevant to determine the effect of 4MU on these biological processes. For this purpose, wound healing and zymography assays were performed.

**Fig. 5:**
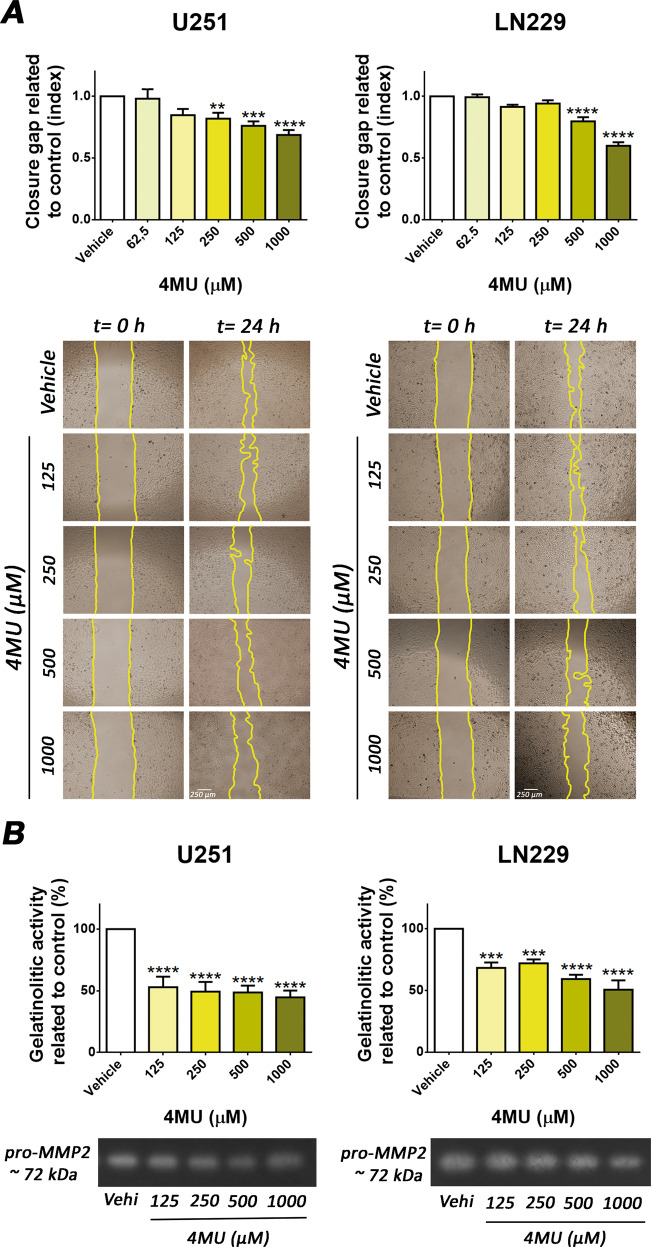
Effect of 4MU on cell migration and MMP activity. **A** U251 and LN229 cell lines were treated with 4MU for 24 h and cell migration was determined by the wound healing assay. The same wound area was photographed at 0 and 24 h. Results are expressed as closure gap index (n = 5) calculated as described in the Material and Methods section. Representative images of independent assays are shown under each bar graph (magnification: ×40). **B** MMPs activity of U251 and LN229 cells after 24 h of treatment with 4MU determined by zymography. The gelatinolytic activity was calculated as the percentage (n = 4) of densitometry values of bands in relation to vehicle control cells. Representative images of independent assays are shown under each bar graph. In all graphs, bars represent means ± SEM of at least three independent experiments. Asterisks represent statistical significance in relation to cells treated with vehicle: ** = *p* < 0.01, *** = *p* < 0.001 and **** = *p* < 0.0001.

As shown in Fig. 5A, after 24 h of treatment, 4MU decreased the wound closure in a dose-dependent manner on both cell lines. Furthermore, at the same time point, 4MU reduced the activity of MMP-2, reaching about 50% of reduction for most doses in LN229 and U251 cells (Fig. 5B). Overall, 4MU inhibited two processes closely related to GBM malignancy, cell migration, and MMP-2 activity.

### Exogenous addition of HA only prevents the effect of 4MU on senescence induction

Because of the well-known effect of 4MU on HA synthesis, we next decided to study if the exogenous addition of HA was able to counteract the effects of 4MU on both U251 and LN229 cells. To fulfill this aim, we performed the XTT, BrdU, wound healing, and zymography assays on GBM cells treated with 4MU, HA, or their combinations.

**Fig. 6:**
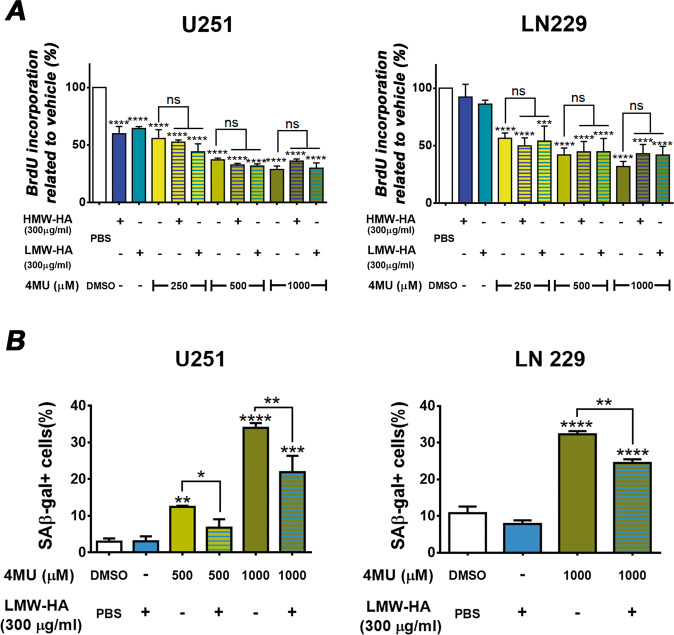
Effect of the co-treatment with 4MU and HA on cell proliferation and senescence induction. **A** U251 (left) and LN229 (right) cells were treated with 4MU plus HMW-HA or LMW-HA for 48 h and cell proliferation was determined by BrdU incorporation and ELISA-like assay. Results are expressed as the percentage (*n* = 3) of cell proliferation in relation to vehicle control cells as described in the Material and Methods section. **B** Both U251 (left) and LN229 (right) cell lines were treated with 4MU plus HMW-HA or LMW-HA for 48 h and senescence induction was determined by SA-β-gal activity assay. Results are expressed as the percentage (*n* = 3) of SA-β-gal positive cells in relation to vehicle control as described in the Materials and Methods section. In all graphs, bars represent means ± SEM of three independent experiments. Asterisks over bars indicate differences between treated and cells treated with vehicle, * = *p* < 0.05, ** = *p* < 0.01, *** = *p* < 0.001, **** = *p* < 0.0001, and ns = non-significant (*p* > 0.05). Symbols over lines indicate differences between the indicated treatments.

As shown in Fig. 6A, the addition of both LMW-HA and HMW-HA failed to counteract the effect of 4MU on cell proliferation in both U251 and LN229 cells. Similar results were observed on metabolic activity, gap closure, as well as MMP-2 activity (Supplementary Fig. 2A–C).

Interestingly, we observed that the exogenous addition of LMW-HA partially prevented the effect of 4MU on senescence induction on both GBM cell lines (Fig. 6B). Similar results were obtained with co-treatment of 4MU with HMW-HA in both cell lines (data not shown).

These findings suggest that the 4MU-induced senescence was partially mediated by the reduction of HA levels. However, the other 4MU effects would be independent of HA synthesis inhibition.

### 4MU downregulates CD44 expression and modifies RHAMM and p-ERK distribution in both cell lines

Since HA-induced migration seems to depend on CD44, RHAMM, and MEK/ERK pathway and that 4MU inhibited such process in a HA-synthesis inhibition independent manner, we evaluated if 4MU modulates the expression, as well as the cellular distribution of these molecules, by WB, FC and/or IF.

**Fig. 7:**
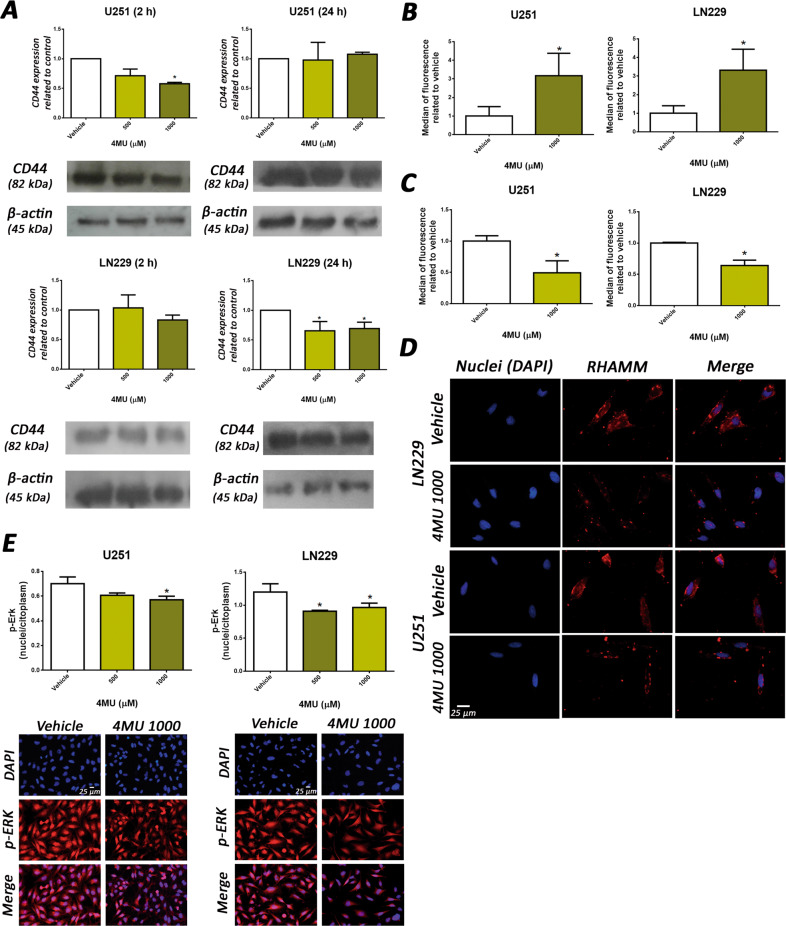
Effect of 4MU on CD44, RHAMM and p-ERK expression. **A** U251 (left) and LN229 (right) cells were treated with 4MU for 2 and 24 h and CD44 total expression was determined by WB. The results are expressed as described in the Materials and Methods section. Both cell lines were treated with 4MU for 24 h and RHAMM total expression **B** and RHAMM membrane expression **C** were determined by FC. The results are expressed as the median of fluorescence related to vehicle control. **D** RHAMM membrane expression was determined by IF assay on U251 (left) and LN229 (right) cell lines after 24 h of treatment with 4MU. Representative images of independent assays are shown under each bar graph (magnification: ×400). **E** U251 and LN229 cells were treated with 4MU for 24 h and p-ERK expression was determined by IF. The results are expressed as the nucleus/total fluorescence intensity related to vehicle condition. Representative pictures of independent assays are shown under each bar graph (maginification: ×200) In all graphs, bars represent means ± SEM of three independent experiments. Asterisks represent statistical significance in relation to cells treated with vehicle: ** = *p* < 0.01, *** = *p* < 0.001 and **** = *p* < 0.0001.

Figure 7A shows that 4MU diminished CD44 expression after 2 h and 24 h of treatment in the U251 and LN229 cells, respectively. Likewise, the treatment with 4MU for 24 h reduced RHAMM membrane levels while increased total RHAMM expression in both cell lines (Fig. 7B, C). Interestingly, analysis by IF showed that 4MU induced a different membrane distribution pattern of RHAMM with respect to the control condition (Fig. 7D). Regarding p-ERK, 4MU treatment did not modify the p-ERK/ERK index assessed by WB (data not shown) but generated a re-localization of p-ERK decreasing its nuclei/cytoplasm ratio (Fig. 7E).

Overall, 4MU reduced the total expression of CD44 and the membrane expression of RHAMM while induced a redistribution of p-ERK, diminishing its level in the nuclei.

## Discussion

GBM is a highly aggressive neoplasm, being the patient median survival of 14.6 months. Mortality is due to the fast-growing rate and surrounding tissue invasion capacity of tumor cells. Hyaluronan has been widely associated with enhanced proliferation, migration, and invasion in GBM [33]. In this work, we demonstrated that HA enhances GBM cell migration through CD44 and RHAMM. Similar results were obtained by several authors in other tumors, which highlights the importance of the interaction between HA and its receptors in cell migration in GBM [14, 38, 39, 44–47]. Furthermore, we showed that the MEK inhibitor, U0126, abrogated HA-induced migration, restoring the baseline condition levels, in agreement with previous reports that demonstrated the involvement of the MEK/ERK pathway in the HA-induced migration in other tumor cell lines [14, 47–49].

Similar to our previous report in the murine GBM model [26], the augment in cell migration mediated by HA was not accompanied by an increase in MMP-2 activity in human GBM cell lines. Taking into account that MMPs are collagenases and considering that the brain matrix is made up majorly of HA, it was postulated that these enzymes are more involved in other processes such as neoangiogenesis rather than in cell migration [50, 51]. Therefore, the treatment with HA would be enough for cell migration but not for MMPs secretion, which would be stimulated by other signaling pathways. In this context, with high amounts of HA, the activity of hyaluronidases might be relevant [44, 52, 53]. Surprisingly, HA inhibited cell proliferation on U251 cell lines while it did not modify such process on LN229 cells. Furthermore, the XTT assay showed that HA increases the metabolic activity on U251 without affecting that on LN229 cells. In view of these results and considering previous reports [27], we hypothesized that HA might enhance the autophagy process in U251 cells. Therefore, Ab index in the XTT assay (which assesses mitochondrial enzymatic activity) could increase in the presence of autophagic cells, since such cell state exerts an increase in mitochondrial activity while cell proliferation remains inhibit.

4MU has been widely used as an HA synthesis inhibitor. In this work, we observed that the GBM cell lines produced high amounts of HA, and that 4MU was able to partially reduce it.

We also showed that 4MU exerts a marked effect on GBM cell proliferation as was demonstrated both in vitro and in vivo by Tao Yan et al. [27], but the levels of cell death reached after 4MU treatments did not exceed 15%. In search of other tumor suppression mechanism that could be implicated, we showed for the first time that 4MU induces senescence on GBM cell lines. This finding is consistent with our previous work showing that 4MU induces senescence in a chronic myeloid leukemia model [13, 54]. Furthermore, senescence has been proposed as an anti-tumor mechanism in GBM and even the treatment with TMZ generates senescence on several GBM cells [55–57].

Although HA-synthesis inhibition might explain, in part, the results of 4MU treatment on GBM cells, we demonstrated that the exogenous addition of HA was not able to counteract 4MU effects on cell growth. Similar results were shown in our previous work using a mouse GBM model [26]. Conversely, Tao Yan et al showed that HA effectively counteracts the effect of 4MU on cell viability [27]. Such discrepancy would be explained by the differences in the methodology as well as the HA concentration and molecular weight used. In the mentioned work the authors assessed only one dose of 4MU (not reported) and HA (25 µg/ml) of non-declared molecular weight, which hinders the comparisons with our results. Furthermore, the authors employ MTT assay for cell viability while in our work we used both XTT and BrdU incorporation assays.

Likewise, we observed that HA did not prevent the effect of 4MU on metabolic and MMP-2 activity. In the same way, several authors proposed HA-independent mechanisms for 4MU effects [13, 24, 25].

Interestingly, exogenous HA was able to partially prevent the senescent effect of 4MU in GBM cell lines. Such results are in agreement with previous reports in K562 cells, as well as in a fibroblast model, in which the inhibition of HAS-2 using miR-23ª-3p led to induction of senescence, demonstrating the anti-senescent effects of HA [58].

Regarding cell migration, we showed for the first time that 4MU inhibits the wound closure in a HA-independent manner in both cell lines, providing data to understand its mechanisms of action. We observed that 4MU markedly reduced GBM cell migration and that the addition of HA did not counteract its effect. As HA-induced migration was dependent on CD44 and RHAMM receptors and MEK/ERK pathway, it was of interest to evaluate the effect of 4MU on the mentioned proteins.

We observed that 4MU diminishes CD44 expression in both GBM cell lines, which is consistent with a previous report in a malignant pleural mesothelioma model [59]. Interestingly, 4MU also reduced RHAMM membrane expression and altered its distribution. Several reports indicate that CD44 and RHAMM are associated with GBM malignant features and poor prognosis [36–38, 47, 60, 61]. Thus, the fact that 4MU reduces the total expression of CD44 and the levels of RHAMM in the cell membrane constitutes a relevant finding, which places 4MU treatment as a promising therapeutic alternative.

At this point, we suggest that the effect of 4MU on cell migration could be caused by the downregulation of HA receptors, mainly RHAMM. Therefore, the addition of exogenous HA cannot counteract 4MU effects because CD44 and RHAMM are not available for interaction at the cell membrane.

Furthermore, we found that 4MU increased RHAMM levels in the intracellular compartment. In this way, optimal levels of RHAMM are required for correct mitosis, since its augment was correlated with the aberrant formation of the mitotic spindle and genomic instability [60, 62]. Therefore, the intracellular increase of RHAMM caused by 4MU might explain not only the effect on cell proliferation but also on senescence induction on the GBM cell lines. Moreover, the 4MU-induced p-ERK redistribution reinforces this point, since the reduction of p-ERK level in the nucleus would also be associated with inhibition of proliferation and probably with senescence induction [63–65]. Furthermore, therapy targeting the translocation of p-ERK from the cytoplasm to the nucleus generates resistance at longer times respect to classical ERK-phosphorylation inhibition, which represents an improvement in cancer therapy [66].

In summary, all these results highlight 4MU anti-tumor effects on human GBM cells and allow us to understand 4MU mechanisms independent of HA-synthesis inhibition, which could represent significant advances in cancer research and in the development of alternative therapies for GBM.

## Methods and materials

### Reagents

Recombinant high molecular weight (HMW, 1.5–1.8 × 10^6^ Da) and low molecular weight (LMW, 1–3 × 10^5^ Da) HA were supplied by Farmatrade (Buenos Aires, Argentina). 4MU, propidium iodide (PI), fluorescein diacetate (FDA), biotinylated Hyaluronic Acid Binding Protein (bHABP), 4′,6-diamidino-2-phenylindole (DAPI), gelatin, glucose, BSA, XTT, X-gal and phenazine methosulfate (PMS) were purchased from Sigma-Aldrich (Saint Luis, Missouri, USA). DMEM, L-glutamine, streptomycin and penicillin were purchased from Invitrogen (Waltham, Massachusetts, USA). The rat antibody anti-CD44 (monoclonal, IM7, ATCC) was produced in our laboratory from the hybridoma. The anti-RHAMM (ab170527) antibody was purchased from Abcam (Cambridge, UK). BrdU, monoclonal mouse anti-BrdU (317902) antibody, goat anti-mouse HRP (405306) secondary antibody were purchased from Biolegend (San Diego, CA, USA). The anti-p-ERK (Tyr204-R, sc-101761) and β-actin (C11, sc-1615) antibodies and horseradish peroxidase-labeled anti-rabbit (sc-2030) and anti-goat (sc-2033) secondary antibodies were from Santa Cruz (Dallas, Texas, USA). The goat anti-mouse cy3 (115-165-003) and the goat anti-rat cy3 (112-165-003) secondary antibodies were obtained from Jackson Immunoresearch (West Grove, Pennsylvania, USA), the donkey anti-rabbit Alexa 594 (R37119), and 488 (R37118) secondary antibodies were purchased from Molecular Probes (Eugene, Oregon, USA) and Mowiol (Calbiochem) was purchased from Merck S.A (Buenos Aires, Argentina).

### Cell cultures

The LN229 and U251 human GBM cell lines (gently provided by Dr. C. Perez-Castro and Dr. M. Candolffi, respectively) were grown in adherent cultures at 37 °C in a 5% CO_2_ atmosphere with DMEM supplemented with 10% heat inactivated fetal bovine serum (FBS), 2 mM L-glutamine, 100 µg/mL streptomycin and 100 IU/mL penicillin (DMEM-C) and tested for Mycoplasma every three months by DAPI staining. Cells passages lower than 20 were used for the described experiments.

### Cell treatments

For all assays, cells were seeded 24 h before treatment. Cells were treated with either HMW-HA or LMW-HA (0–300 µg/ml), or 4MU (0–1000 µM), or a combination of them as appropriate. Untreated control cultures were also included. The anti-RHAMM (1/100) and anti-CD44 (1/50) antibodies were used to block these receptors while U0126 (10 µM) was used to inhibit MEK. All incubations were performed at 37 °C in a 5% CO_2_ atmosphere.

### XTT assay

For the XTT assay, 5 × 10^3^ cells/well were seeded in 96-well plates and treated with 4MU, and/or HA for 48 h. After treatment, 25 μl of an XTT solution (1 mg/ml) containing PMS (7.5 µg/ml) were added to the culture medium (100 μl) and cells were incubated for two additional hours at 37 °C in a 5% CO_2_ atmosphere. After incubation, the absorbance (Ab) was read at 450 nm and 620 nm using a microplate reader (Multiscan Ex, Absorbance Microplate Reader, Thermo Electron Corporation, China). Cell viability was calculated as

$${\rm{Ab}}\,{({\rm{treated}})_{450}} - {\rm{Ab}}\,{({\rm{treated}})_{620}}/{\rm{Ab}}\,{({\rm{untreated}})_{450}} - {\rm{Ab}}\,{({\rm{untreated}})_{620}}$$

### Cell proliferation

Cell proliferation was determined by BrdU incorporation and ELISA-like and IF assays.

*IF:* Briefly, 1.5 × 10^4^ cells/well were seeded in 24-well plates on coverslips and treated for 47.5 h with 4MU, HA or their combinations. Then, BrdU was added at final concentration of 10 µM and cells were incubated for 30 min. After this time, the supernatant was removed, the cells were fixed with PFA 4% by 20 min and permeabilized with HCl 2 N. Then, cells were neutralized with sodium tetraborate (0.1 M; pH = 9), blocked with SFB 2% O.N at 4 °C and incubated with the mouse anti-BrdU antibody O.N. at 4 °C. Finally, the anti-mouse secondary antibody was added plus DAPI (1 µg/ml) and incubated for 2 h. The coverslips were mounted with Mowiol and observed by fluorescence microscopy. Proliferation was calculated as:

$$[{\rm{BrdU}} \, {\rm{positive}} \, {\rm{nuclei}}/{\rm{total}} \, {\rm{of}} \, {\rm{nucleus}}] \times 100$$

*ELISA-like:* Briefly, 3 × 10^3^ cells/well were seeded in 96-well plate and treated for 46.5 h with 4MU, HA or their combinations. Then, BrdU was added at final concentration of 10 µM and cells were incubated for 1.5 h. After this time, the supernatant was removed, the cells were washed and fixed with PFA 4% for 20 min and permeabilized with HCl 2 N. Then, cells were neutralized with sodium tetraborate (0.1 M; pH=9) and the endogenous peroxidase activity was blocked with 3% H_2_O_2_ in methanol for 30 min at room temperature. After that, cells were blocked with SFB 2% O.N. and incubated with the mouse anti-BrdU antibody (1/1000) O.N. at 4 °C. Finally, the HRP conjugated anti-mouse antibody was added (1/2000) and incubated for 2 h at room temperature. The plate was revealed with TMB and reaction stopped after 10 min with 4 N H_2_SO_4_ and absorbance was read at 450 nm and 620 nm using a microplate reader (Multiscan Ex, Absorbance Microplate Reader, Thermo Electron Corporation, China). Cell proliferation was calculated as:

$$[{\rm{Ab}}\,{({\rm{treated}})_{450}} - {\rm{Ab}}\,{({\rm{treated}})_{620}}/{\rm{Ab}}\,{({\rm{untreated}})_{450}} - {\rm{Ab}}\,{({\rm{untreated}})_{620}}] \times 100$$

### Cell death

Propidium iodide staining was performed, as previously described [13, 67–69]. Briefly, 3 × 10^5^cells/well were seeded in 12-well plates and treated for 72 h. Cells were then stained with FDA (1.4 µM) for 20 min, harvested, centrifuged, and washed. Subsequently, the cell pellet was resuspended and incubated with PI (5 μg/ml) for 5 min. Stained cells were acquired on a Pas III flow cytometer (Partec, Germany) and analyzed with the Flowing 2.1.5 software (Scripps Institute, La Jolla, USA).

### Evaluation of senescence

Cell senescence was assessed through SA-β-gal activity [70] and the presence of cytoplasmic chromatin fragments (CCF) [71]. For SA-β-gal, cells (5 × 10^5^ cells/ml) were seeded on coverslips in 24-well plates and incubated with either DMEM-C, 4MU, HA or their combinations at 37 °C in a 5% CO_2_ atmosphere. After 48 h, cells were fixed with PBS plus 2% PFA and washed with PBS. Then, fixed cells were incubated for 24 h at 37 °C with staining solution (1 mg/ml X-gal, 5 mM potassium ferricyanide, 5 mM potassium ferrocyanide, 2 mM MgCl_2_, 150 mM NaCl, 30 mM citric acid/phosphate pH = 6). Cells were washed twice with PBS and the SA-β-gal activity was evaluated in an Olympus BX51 (America Inc.) microscope. Blue cells were considered positive. For each condition, 300 cells were counted and the percentage of SA-β-gal positive cells was calculated [70, 72]. For CCF, cells were subjected to similar treatments. After 48 h, cells were fixed with PBS plus 2% PFA, washed and incubated with 1 μg/ml DAPI in PBS plus 0.2% Triton X-100 for 20 min at room temperature. Cells were analyzed by fluorescence microscopy (Olympus BX51, America Inc.).

### Measurement of HA levels by enzyme-linked immunosorbent assay

HA levels in cell supernatant were measured by a competitive enzyme-linked immunosorbent assay (ELISA), as described previously [26]. Briefly, 96 well microtiter plates were coated with 100 µg/ml HMW-HA at 4 °C. Samples and standard HMW-HA were incubated with 0.75 µg/ml bHABP at 37 °C. The plate was blocked and incubated with the samples at 37 °C for 4 h. After washing, the bHABP bound was determined using an avidin–biotin detection system. Sample concentrations were calculated from a standard curve.

### Zymography

The MMPs activity was evaluated by gelatin zymography as previously described [73, 74]. Briefly, 2 × 10^5^ cells/well were incubated in 48-well plates with serum-free DMEM supplemented with 0.01% BSA and 0.1% glucose with or without each treatment. After 24 h, supernatants were centrifuged and loaded on 7.5% SDS-PAGE gels containing gelatin (1 mg/ml). A molecular weight marker was run in parallel. After electrophoresis, gels were washed three times with 2.5% Triton X-100 for 20 min, and then incubated with 25 mM Tris-HCl pH 7.5; 5 mM CaCl_2_; 0.9% NaCl; 0.05% NaN_3_ for 48 h at 37 °C. The gelatinolytic activity was revealed by staining with 0.5% Coomassie blue. Photographs were obtained with a BioSpectrum® 515 Imaging System M-26XV (UVP, Cambridge, UK) and analyzed with the Image J software.

### Wound healing assay

Migration assay was performed as previously described, with slight modifications [75]. Cells were seeded in 24-well plates until they reached confluence. The monolayer was then scratched with a 200 µl sterile pipette tip and incubated in DMEM containing 3% SFB with or without each treatment. The same wound area was photographed at 24 h. The Image J software was used to calculate wound area.

Results were expressed as migration index calculated as

$${\rm{Closure}} \, {\rm{gap}} \, {\rm{index}}=[({\rm{area}})_{t=0h}-({\rm{area}})_{t=24h}]_{\rm{treated}}/[({\rm{area}})_{t=0hs}-({\rm{area}})_{t=24h}]{\rm{control}}$$

### Evaluation of CD44, RHAMM and ERK/p-ERK

The receptors and ERK/p-ERK were evaluated by IF, western blot (WB), and/or flow cytometry as mentioned below:

*IF:* This assay was performed as previously described, with slight modifications [26]. Briefly, after each treatment, cells were fixed with 4% PFA in PBS for 20 min, washed and blocked with PBS containing 5% FSB and 0.1% Triton X-100 for 2 h. Cells were then incubated in 1% FBS overnight at 4 °C with the following primary antibodies: anti-CD44 clone IM7 (1/50), anti-RHAMM (1/100), anti-p-ERK (1/100), and 1 µg/ml DAPI dye. After washing, cells were incubated in 1% FBS for 2 h at room temperature with the following secondary antibodies: goat cy3 anti-rat (1/500), donkey anti-rabbit Alexa 594 (1/2000), donkey anti-rabbit Alexa 488 (1/2000), and 1 µg/ml DAPI dye. Then, the coverslips were mounted with Mowiol mounting medium on glass slides and micrographs were obtained by an Olympus BX51 microscope equipped with an Olympus DP73 camera (Olympus America Inc). Images were analyzed with the FIJI software (media Cybernetics). The same procedure was performed without permeabilization to evaluate the membrane expression of RHAMM.

*WB:* This assay was performed as previously described [13, 69]. Briefly, after treatments for 2 or 24 h at 37 °C in a 5% CO_2_ atmosphere, cells were lysed with hypotonic buffer, centrifuged (13,000 rpm 30 min) and equal amounts of protein were resolved by SDS-polyacrylamide gel electrophoresis and transferred onto a PVDF membrane (Osmonics Inc., Gloucester, MA). The membrane was blocked and incubated with specific antibodies to RHAMM (1/100), CD44 (1/500), p-ERK (1/500), ERK (1/500), or β-Actin (1/1000) overnight at 4 °C followed by incubation with a horseradish peroxidase-labeled secondary antibody for 2 h at 37 °C. The reaction was developed using a chemiluminescent detection system. Gel images obtained with a digital camera were subjected to densitometric analysis using the Image Scion Software (Scion Corporation, USA). The results are expressed as:

$$({\rm{Interest}} \, {\rm{protein}}/{\rm{actin}})_{\rm{tto}}/({\rm{Interest}}\, {\rm{protein}}/{\rm{actin}})_{\rm{vehicle}}$$

*Flow cytometry:* 3 × 10^5^cells/well were seeded in 12-well plates and treated for 24 h. After that, cells were harvested, centrifuged, washed twice with PBS and fixed for 15 min with 2% paraformaldehyde (PFA). Then, Fc receptors on cells were blocked with 2% normal human serum (NHS) in PBS for 30 min and cells were incubated with anti-RHAMM (1/100) for 1 h at 4 °C, followed by the addition of anti-rabbit secondary antibody conjugated with Alexa 647 (1/1000) for 30 min at 4 °C in the dark. This procedure was performed with or without cell permeabilization to evaluate total or membrane RHAMM, respectively. In order to control unspecific signals, cells were incubated only in the presence of secondary antibody. Stained cells were acquired on a Pas III flow cytometer (Partec, Germany) and analyzed with the Flowing 2.1.5 software (Scripps Institute, La Jolla, USA).

### Statistical analysis

Statistical significance was calculated using Prism 6 statistical software (Graph Pad Prism, San Diego, CA, USA). The data presented in this study are expressed as mean values ± SEM if not otherwise stated. The letter “n” refers to the number of independently performed experiments representative of the data shown in the figures. This number was selected in each case considering the statistic test and the possibilities of the laboratory and it was shown in the captions of each figure. Shapiro–Wilk´s normality test was performed prior to statistical test. The variances were analyzed within each group of data by Levene’s test and were similar between the groups that are being statistically compared. One way-ANOVA was used to compare three or more independent groups. The Student’s test (T-test) was performed to compare two independent groups while to compare treated vs. untreated groups Dunnet’s test was performed. Bonferroni’s test was used in order to make simultaneous multiple comparisons between different groups. *P* values < 0.05 were considered statistically significant.

## Supplementary information

Fig. Sup. 1

Fig. Sup. 2
