# Supplementary figures and images for: Antitumor effect of 4MU on glioblastoma cells is mediated by senescence induction and CD44, RHAMM and p-ERK modulation
Source: Cell Death Discov. 2021 Oct 9;7:280. doi: 10.1038/s41420-021-00672-0 (PMC8502173; doi:10.1038/s41420-021-00672-0)

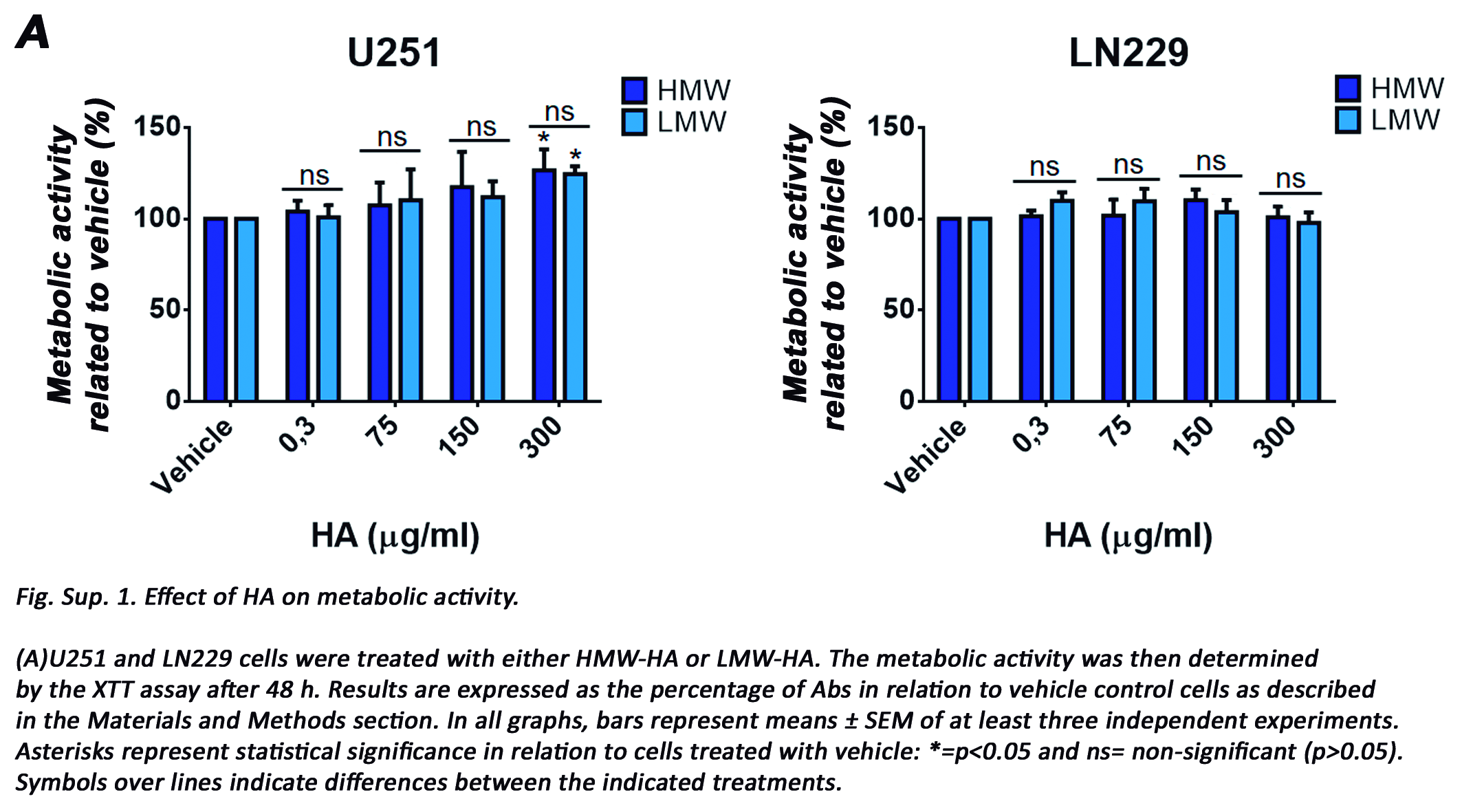

Supplement: Supplementary file 1 — Fig. Sup. 1 [file 41420_2021_672_MOESM1_ESM.tif]

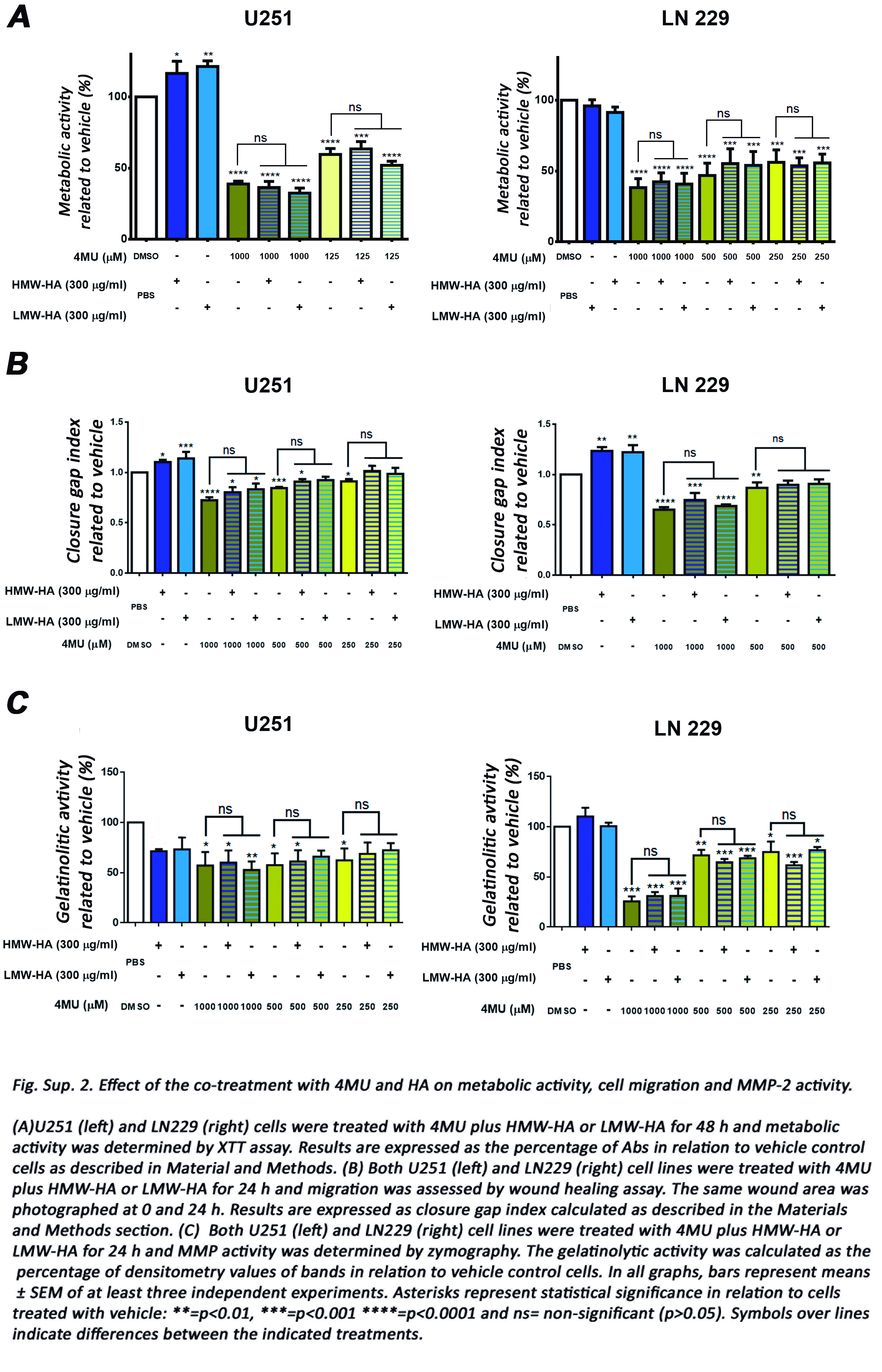

Supplement: Supplementary file 2 — Fig. Sup. 2 [file 41420_2021_672_MOESM2_ESM.tif]
